# Supplementary material for: Geometry symmetry-free and higher-order optical bound states in the continuum
Source: Nat Commun. 2021 Jul 19;12:4390. doi: 10.1038/s41467-021-24686-5 (PMC8290025; doi:10.1038/s41467-021-24686-5)
Supplement: Supplementary file 1 — Supplementary information for Geometry symmetry-free and Higher-order Optical Bound States in the Continuum [file 41467_2021_24686_MOESM1_ESM.pdf]

Supplementary information for

Geometry symmetry-free and Higher-order Optical Bound States in the Continuum

Qingjia Zhou[1, 2], Yangyang Fu[3], Lujun Huang[4], Qiannan Wu[5], Andrey Miroshnichenko[4], Lei Gao[1, 2] and Yadong Xu[1, 2, 6]

[1]School of Physical Science and Technology & Collaborative Innovation Center of Suzhou Nano Science and Technology, Soochow University, Suzhou 215006, China.

[2]Jiangsu Key Laboratory of Thin Films, Soochow University, Suzhou 215006, China.

[3]College of Science, Nanjing University of Aeronautics and Astronautics & Key Laboratory of Aerospace Information Materials and Physics (NUAA), MIIT, Nanjing 211106, China.

[4]School of Engineering and Information Technology, University of New South Wales, Canberra, ACT, 2600 Australia.

[5]School of Science, North University of China, Taiyuan, Shanxi, 030051, China.

[6]State Key Laboratory of Functional Material for Informatics, Shanghai Institute of Microsystem and Information Technology, Chinese Academy of Sciences, Shanghai 200050, China.

This supplementary information includes:

Supplementary Notes 1 to 5

Supplementary Figures 1 to 11

Supplementary References

Supplementary Note 1. Eigenmode analysis of ZIM with objects

In simulation: Full-wave simulations were carried out by using software COMSOL Multiphysics to study the eigenfrequency and eigenmode profiles. In Fig. 2, the working frequency is 15 GHz, and the linewidth vanishes at $\alpha = 0$ and $\varepsilon_{\rm d} = 4.82$. For eigenmode analysis, we studied the case of a ZIM background containing two same objects with $\varepsilon_{\rm d} = 4.82$ instead. The infinite ZIM is replaced by a large ZIM area under a scattering boundary condition. We obtain two eigenfrequencies and the corresponding field patterns as shown in Fig. 3b. For the case of two cylinders with $\varepsilon_{\rm d} = 4.82$ and $R_1 = R_2 = 8\ \text{mm}$ embedded in ZIM environment, the system supports a bright mode and a dark mode (i.e., BIC mode, at about 15 GHz). The small deviation is mainly due to finitely large ZIM area and the accuracy of grid divisions in simulations. Similar procedures were applied to the case of three cylinders with $\varepsilon_{\rm d} = 4.82$ and $R_1 = R_2 = R_3 = 8\ \text{mm}$ embedded in ZIM environment. Based on numerical calculations, we can easily get the eigenfrequency of each eigenmode and its corresponding field pattern, as shown in Fig. 4b.

Theoretical analysis: It is evident from Eq. (1) that the eigenfrequency of studied system also can be strictly calculated by setting the denominator term of the Eq. (1) to be zero, that is $1 - i(\pi/w)\sum_{i=1}^{N} R_i J_1\left(\sqrt{\varepsilon_{\rm d}} k_0 R_i\right) \Big/ \sqrt{\varepsilon_{\rm d}} J_0\left(\sqrt{\varepsilon_{\rm d}} k_0 R_i\right) = 0$. By solving this equation with considered geometric parameters, a complex eigenfrequency $\omega = \omega_0 - i\gamma$ can be obtained analytically, and the Q factor of the corresponding eigenmode is defined by $Q = \omega_0 / 2\gamma$. Based on this method, Supplementary Fig. 2a analytically shows the calculated eigenfrequency that decreases with the increase of α. In particular, at $\alpha = 0$, the eigenfrequency is 15 GHz, which is marked with black star in plot. In Supplementary Fig. 2b, the Q factor turns to be infinite at $\alpha = 0$, where means such a mode is BIC mode. These analytical results are consistent with those based on numerical simulations. Similarly, the cases of N=3 and N=5 were also analyzed, with the obtained results shown in Supplementary Fig. 3a. It

is found that Q still trends to infinity at $\alpha = 0$ for N=3 or N=5. For the same $\alpha \neq 0$, the Q factor of the quasi-BIC is larger for a larger N. In addition, the results also indicate that the Q factor is inversely proportional to the square of α (see Supplementary Fig. 3b). These results further confirm the existence of higher-order BICs.

Supplementary Note 2. Analysis of BIC in the ZIM embedded with a rectangular object and a cylindrical object with different materials

According Eq. (1) in the main text, if a rectangular object with permittivity ε_{r} and a cylinder with permittivity ε_{c} embedded in the ZIM (see Supplementary Fig. 8a), the transmission coefficient can be written as

$$T = \frac{1}{1-\left(i\omega/2wH_1\right)\left[\oint_{\partial C_{\mathrm{r}}}\mathbf{A}_{\mathrm{r}}\cdot d\mathbf{l}+\oint_{\partial C_{\mathrm{c}}}\mathbf{A}_{\mathrm{c}}\cdot d\mathbf{l}\right]}, \tag{1}$$

where $\mathbf{A}_{\mathrm{r}}$ is the magnetic potential at the boundary ∂C_{r} of the rectangular object and $\mathbf{A}_{\mathrm{c}}$ is the magnetic potential at the boundary ∂C_{c} of the cylinder. Further, the magnetic flux inside the cylinder is

$$\varphi_c = \oint_{\partial C_c}\mathbf{A}_c\cdot d\mathbf{l} = \frac{2\pi H_1}{\omega}\frac{J_1\left(k_c R\right)R}{J_0\left(k_c R\right)\sqrt{\varepsilon_c}}, \tag{2}$$

where $k_{\mathrm{c}} = \sqrt{\varepsilon_{\mathrm{c}}}\,\omega/c$, ε_{c} is the permittivity of the cylinder and *R* is the radius of the cylinder. The magnetic flux inside the rectangular object is

$$\varphi_r = \oint_{\partial C_r}\mathbf{A}_r\cdot d\mathbf{l} = \frac{1}{i\omega}\oint_{\partial C_r}\mathbf{E}_r\cdot d\mathbf{l}. \tag{3}$$

Here $\mathbf{E}_{\mathrm{r}}$ is electric field in the rectangular object [1], given by

$$\mathbf{E}_{\mathrm{r}} = \frac{i}{\omega\varepsilon_0\varepsilon_{\mathrm{r}}}\left(\hat{\mathbf{x}}\sum_{n_x,n_y\geq 1}C_{n_x,n_y}\frac{\partial\phi_{n_x,n_y}}{\partial y}-\hat{\mathbf{y}}\sum_{n_x,n_y\geq 1}C_{n_x,n_y}\frac{\partial\phi_{n_x,n_y}}{\partial x}\right), \tag{4}$$

with

$$C_{n_x,n_y} = \frac{-4k_{\mathrm{r}}^2 H_1\left[1-(-1)^{n_x}\right]\left[1-(-1)^{n_y}\right]}{n_x n_y\left[k_{\mathrm{r}}^2-\left(n_x\pi/a\right)^2-\left(n_y\pi/b\right)^2\right]\pi^2}, \tag{5}$$

where $k_{\mathrm{r}} = \sqrt{\varepsilon_{\mathrm{r}}}\,\omega/c$, ε_{r} is the permittivity of the rectangular object, *a* and *b* represent rectangle side length, n_x (n_y) is non-zero positive integer number for indicating the standing waves in *x*(*y*) direction, and $\phi_{n_x,n_y} = \sin\frac{n_x \pi x}{a}\sin\frac{n_y \pi y}{b}$.

Next, we will show that this system also supports BIC when the two objects are filled with different materials. We assume that the ZIM is still non-dispersive and consider BIC in frequency domain. The relevant parameters are: *a*=6 mm, *b*=4.01 mm, $\varepsilon_{\mathrm{r}} = \varepsilon_{\mathrm{d1}} = 9$, and $\varepsilon_{\mathrm{c}} = \varepsilon_{\mathrm{d2}} = 4.82$. The analytical transmission vs the operating frequency and *R* is shown in Supplementary Fig. 8b. The illustrated results are similar with those of Fig. 2a in the main text. A vanishing linewidth appears at *f*=15 GHz and *R*=8.00 mm. Supplementary Fig. 8c shows the corresponding eigenmode analysis, which reveals that a radiation state and a BIC mode are degenerated at 15 GHz. Supplementary Fig. 8d shows transmission spectrum for *R*=8.10 mm. The analytical results agree well with simulated results. The first dip results from monopole mode resonance inside the cylinder, which corresponds to a zero of the Bessel function, i.e., $J_0(k_{\mathrm{c}}R) = 0$. The second dip results from monopole mode resonance inside the rectangular object, which corresponds to the vanishing of denominator in Supplementary Eq. (5), i.e., $k_{\mathrm{r}}^2 - (\pi/a)^2 - (\pi/b)^2 = 0$. For transmission peak, the monopole mode resonances happen inside two objects, and they are out of phase. When *R*=8.00 mm, two monopole modes are degenerated at 15 GHz, leading to a vanishing linewidth.

Supplementary Note 3. Analytical derivation of *Q* factor of quasi-BIC resonances

To calculate the Q factor in Fig. 4c, we define it as $Q = n_{\max} / \Delta n$ ($n = \sqrt{\varepsilon_{\mathrm{d}}}$), where $n_{\max}$ is the refractive index corresponding to transmission peak and Δn is the full width half maximum bandwidth (FWHM). The relative permittivity of all cylinders are the same, i.e. ε_{d}, and the wave vector in cylinders is k_{d}. According to Eq. (1) and Eq. (2) in the main text, the transmission coefficient

$$T = \frac{1}{1 - iX}, \tag{6}$$

with

$$X = -\frac{\pi}{w} \sum_{i=1}^{N} \frac{R_i J_1(k_{\mathrm{d}} R_i)}{\sqrt{\varepsilon_{\mathrm{d}}} J_0(k_{\mathrm{d}} R_i)}. \tag{7}$$

As mentioned in the main text and shown in Supplementary Fig. 9, if all cylinders have different radii (i.e., $i \neq j, \alpha_i \neq \alpha_j$), N cylinders will lead to N-1 transmission peaks with N-1 different Q factors. It is difficult to get the expression of each Q factor in a general case. Here in order to explore the relationship between N and Q factor, we consider a simple case: N-1 cylinders with radius R_{a} and only a cylinder with radius R_{b}. In this case, the system supports (N-2)-fold degenerate BICs, then only one transmission peak in transmission spectrum is left. Next, we will derive the Q factor of this transmission peak. In this case, Supplementary Eq. (7) can be written as

$$X = \frac{\pi}{w} \left[-(N-1) \frac{R_{\mathrm{a}} J_1(k_{\mathrm{d}} R_{\mathrm{a}})}{\sqrt{\varepsilon_{\mathrm{d}}} J_0(k_{\mathrm{d}} R_{\mathrm{a}})} - \frac{R_{\mathrm{b}} J_1(k_{\mathrm{d}} R_{\mathrm{b}})}{\sqrt{\varepsilon_{\mathrm{d}}} J_0(k_{\mathrm{d}} R_{\mathrm{b}})} \right]. \tag{8}$$

Further, we set $x = \sqrt{\varepsilon_{\mathrm{d}}}$ (refractive index), then $k_{\mathrm{d}} R_{\mathrm{a(b)}} = x k_0 R_{\mathrm{a(b)}}$. Firstly, we find the permittivity of the transmission valleys, which corresponds to the solution of $J_0(s_{\mathrm{v}}) = 0$. In this work, the considered parameters lead to s_2=5.52, which means $x_{\mathrm{a}} = s_v / k_0 R_{\mathrm{a}}$ and $x_{\mathrm{b}} = s_v / k_0 R_{\mathrm{b}}$. Because there are only two different sizes of all cylinders in the system, the asymmetry parameter is defined by $\alpha = (R_{\mathrm{b}} - R_{\mathrm{a}})/R_{\mathrm{a}}$. For $\alpha \in (0.99, 1.01)$, the value of $|x_{\mathrm{a}} - x_{\mathrm{b}}|$ is very small, then Taylor series of the

Bessel functions around x_a and x_b are used:

$$J_0(xk_0R_a) \approx J_0(x_ak_0R_a) - k_0R_aJ_1(x_ak_0R_a)(x-x_a),$$
$$J_0(xk_0R_b) \approx J_0(x_bk_0R_b) - k_0R_bJ_1(x_bk_0R_b)(x-x_b). \quad (9)$$

Substituting Supplementary Eq. (9) to Supplementary Eq. (8) and using $J_1(xk_0R_a) \approx J_1(x_ak_0R_a)$, we get

$$X = \frac{\pi}{wk_0x}\left(\frac{N-1}{x-x_a} + \frac{1}{x-x_b}\right). \quad (10)$$

When $|T| = 1/2$, combining Supplementary Eq. (6) and (10) leads to the following equation,

$$\left(\frac{N-1}{x-x_a} + \frac{1}{x-x_b}\right) = \pm\frac{\sqrt{3}wk_0x}{\pi}. \quad (11)$$

In Supplementary Eq. (11), the right-hand side of the equation is approximately equal to $\pm\sqrt{3}wk_0(x_a+x_b)/2\pi$. For easy analysis, we set

$$g_1 = \frac{\sqrt{3}wk_0}{\pi}\left(\frac{x_a+x_b}{2}\right), \quad g_2 = -\frac{\sqrt{3}wk_0}{\pi}\left(\frac{x_a+x_b}{2}\right).$$

With g_1 and g_2, the solutions of Supplementary Eq. (11) are

$$x_2^{(1)} = \frac{x_a+x_b}{2} + \frac{N}{2g_1} - \frac{\sqrt{g_1^2(x_a-x_b)^2 + N^2 + g_1(2N-4)(x_a-x_b)}}{2g_1}, \quad (12a)$$

$$x_2^{(2)} = \frac{x_a+x_b}{2} + \frac{N}{2g_2} - \frac{\sqrt{g_2^2(x_a-x_b)^2 + N^2 + g_2(2N-4)(x_a-x_b)}}{2g_2}. \quad (12b)$$

Because the value of $|x_a - x_b|$ is very small, x_{max} can take an approximation,

$$x_{max} \approx \frac{x_a+x_b}{2}. \quad (13)$$

Finally, the Q factor is defined by $Q = x_{max}/|x_2^{(2)} - x_2^{(1)}|$, and after some simplifications, we have

$$Q = \frac{2N}{(x_a-x_b)^2}\frac{\pi}{\sqrt{3}wk_0}, \quad (14)$$

where $x_a = s_v/(k_0R_a)$ and $x_b = s_v/(k_0R_b)$. Further, substituting x_a and x_b into the Supplementary Eq. (14) produces the following formula,

$$Q = \frac{2\pi k_0R_b^2}{\sqrt{3}ws_v^2}\frac{N}{\alpha^2}. \quad (15)$$

Supplementary Note 4. Effective permittivity and permeability of waveguide junction.

The permittivity is modeled with waveguide dispersion of TE_{10} [2],

$$\varepsilon_{\text{eff}} = \varepsilon_{\text{b}} - \lambda^2/4H^2 , \quad (16)$$

where ε_{b} is the relative permittivity of the medium filling in the waveguide, H is height of waveguide, and λ is the working wavelength in free space. Supplementary Eq. (16) is similar to the Drude model. The red curve in Supplementary Fig. 10 shows the relationship between ε_{eff} and frequency. Clearly, ε_{eff} changes slowly with frequency so that the ENZ window is relatively broadband. After doping a silicon rod into the waveguide junction, the effective permeability of the waveguide junction can be written as [3],

$$\mu_{\text{eff}} = 1 + \frac{A_0}{A}\left[\frac{2}{k_{\text{Si}}^{\text{eff}} R_0} \frac{J_1\left(k_{\text{Si}}^{\text{eff}} R_0\right)}{J_0\left(k_{\text{Si}}^{\text{eff}} R_0\right)} - 1\right], \quad (17)$$

where $A_0 = \pi R_0^2$ is cross-sectional area of silicon rod, $A = L^2 - 3\times16\times\pi r_{\text{w}}^2$ is the total cross-sectional area of air region, r_{w} is the radius of metallic wires, $k_{\text{Si}}^{\text{eff}} = \sqrt{\varepsilon_{\text{Si}}^{\text{eff}}}\,\omega/c$ is the wave vector in silicon, and $\varepsilon_{\text{Si}}^{\text{eff}} = \varepsilon_{\text{Si}} - \lambda^2/4H^2$ is the effective permittivity of silicon for TE_{10} mode. Using Supplementary Eq. (17), we can obtain the relationship between μ_{eff} and frequency, as shown in Supplementary Fig. 10. As showed by the blue curve, μ_{eff} changes quickly with frequency, leading to the ZIM window that has an extremely narrowband response. One may broaden ZIM window by changing the material and shape of the dopant.

Supplementary Note 5. Geometry symmetry-free BIC induced by ENZ medium.

In epsilon near zero (ENZ) medium, the Eq. (1) in the main text can be generalized to [4],

$$T = \frac{1}{1 - \left[ik_0 \mu_1 \left(S - S_{\mathrm{d}} \right) / 2w + \left(i\omega / 2wH_1 \right) \sum_{i=1}^{N} \oint_{\partial C_i} \mathbf{A}_i \cdot d\mathbf{l} \right]} , \tag{18}$$

where $S = l \times w$ and S_{d} is the sum of areas of embedded objects. Likewise, we consider two cylinder objects for illustration and comparison. Supplementary Fig. 11a shows the calculated transmission vs both ε_{d} and α, from which a BIC can be seen at about $\varepsilon_{\mathrm{d}} = 4.82$ and $\alpha = 0$, as indicated by the dashed circle. Supplementary Fig. 11b shows the transmission for $\alpha = -0.01$ case, and for comparison, numerical simulations were carried out using COMSOL with the geometry parameters that are same to those in Fig. 2. The obtained simulated results of two cases of different location distributions are displayed respectively by two kinds of different color balls. Both theoretical results and simulation results agrees well with each other. Similar EIT-like behavior is seen regardless of the locations of the two dielectric rods, and the total transmission indicated by the P corresponds to the quasi-BIC. The two valleys stem from monopole mode resonances that occur in either the left object (see the left pattern in Supplementary Fig. 11c, d) or the right object one (see the right pattern in Supplementary Fig. 11c, d). For the quasi-BIC, due to the term of $\tau = ik_0 \mu_1 \left(S - S_{\mathrm{d}} \right) / 2w$, the magnetic flux induced by two object are not exactly out of phase at all (see the middle pattern in Supplementary Fig. 11c, d). The ENZ leads a modified condition: $\tau + \left(i\pi / w \right) \left(\Phi_1 + \Phi_2 \right) = 0$ and $\Phi_1 \Phi_2 < 0$ for quasi-BIC. Note that the total transmission at about $\varepsilon_{\mathrm{d}} = 5.0$ is not BIC mode, which results from in-phase resonances of EM wave inside two objects, corresponding the condition: $\tau + \left(i\pi / w \right) \left(\Phi_1 + \Phi_2 \right) = 0$ and $\Phi_1 \Phi_2 > 0$. As $\alpha \to 0$, the induced flux Φ_i (i=1, 2) becomes very large, so $\left| \Phi_i \right| \gg \tau$. When $\alpha = 0$, the term of $\tau = ik_0 \mu_1 \left(S - S_{\mathrm{d}} \right) / 2w$ can be negligible compared with Φ_i, so that the ideal BIC happens at $\sum \Phi_i = 0$ and $\Phi_i \neq 0$, which coincides to the results revealed in the main text that the BIC mode is physically

related to the nontrivial zeros of total magnetic flux in all embedded objects.

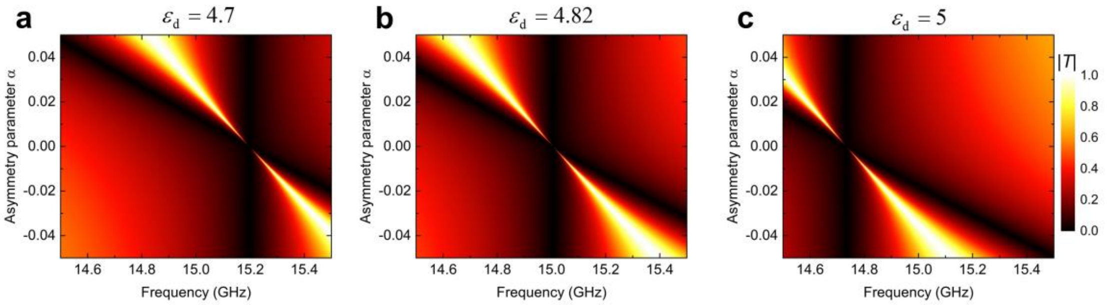

Supplementary Figure 1. The BIC exists for any value of the objects' permittivity as long as the working frequency of BICs is located in the ZIM window. We calculated analytically the transmission coefficient as a function of the working frequency and asymmetry parameter for different permittivity of voids: **a** $\varepsilon_d = 4.7$; **b** $\varepsilon_d = 4.82$; **c** $\varepsilon_d = 5$. In analysis, the considered model is the same as that shown by Fig. 2a in the main text. We can clearly see that the BIC is red-shifted and preserved with the increase of permittivity. The working frequencies of BICs are slightly shifted accordingly, but still located in the frequency window of ZIM, whose bandwidth is about 4%, as displayed in Fig. 5a. Therefore, similar to the conventional symmetry-protected BIC, the ZIM-based BIC also exists at any permittivity of dielectric voids, as long as the working frequency of the proposed BICs is located in the ZIM window.

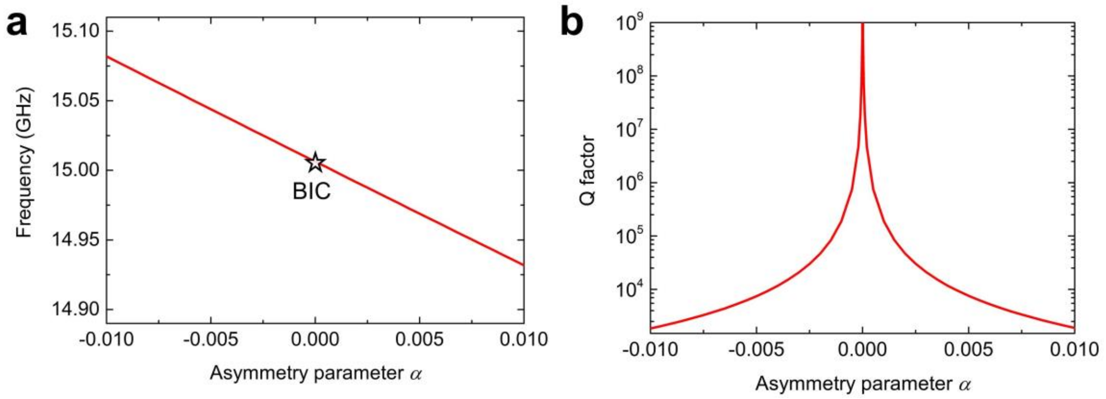

Supplementary Figure 2. Eigenfrequency of studied system for case of *N*=2. a Eigenfrequency as a function of asymmetry parameter. The eigenfrequency is about 15 GHz for $\alpha \in (-0.01, 0.01)$. The eigenmode turns to BIC mode at $\alpha = 0$. **b** Q factor vs asymmetry parameter $\alpha = 0$. The Q factor is infinite at $\alpha = 0$. In analysis, $R_1 = 8$ mm, $\alpha = (R_2 - R_1)/R_1$, $\varepsilon_d = 4.82$ and $w = 44$ mm.

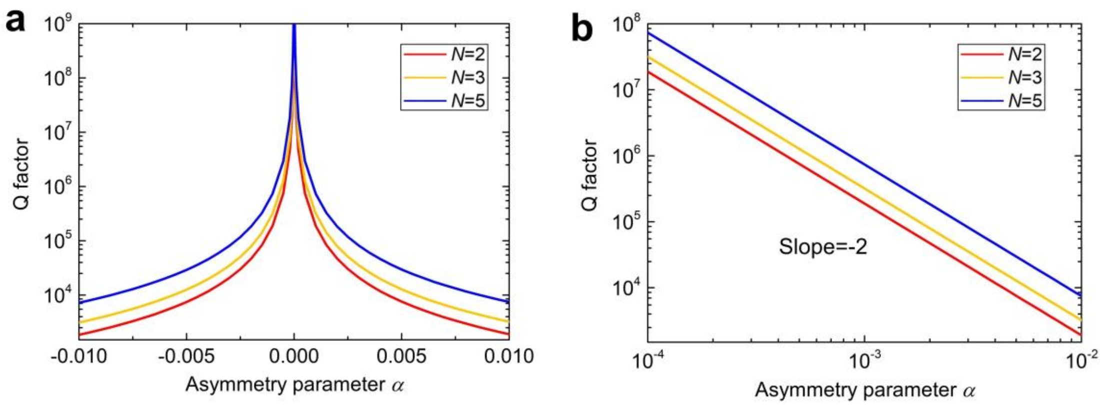

Supplementary Figure 3. High-order BICs. a Q factor of quasi-BICs vs asymmetry parameter $\alpha = 0$ for N=2 (red), N=3 (yellow) and N=5 (blue). Here we consider the case of N objects that include (N-1) identical objects with a fixed radius of R_{a}, and one object with a variable radius of R_{b}. Related parameters are $R_{\mathrm{a}} = 8\ \mathrm{mm}$, $\alpha = \left(R_{\mathrm{b}} - R_{\mathrm{a}}\right)/R_{\mathrm{a}}$, $\varepsilon_{\mathrm{d}} = 4.82$ and $w = 44\ \mathrm{mm}$. **b** Log scale of **a**. N-dependent Q factor of linear shape can be observed. The vary trend of Q factor is consistent with Fig. 4c in the main text.

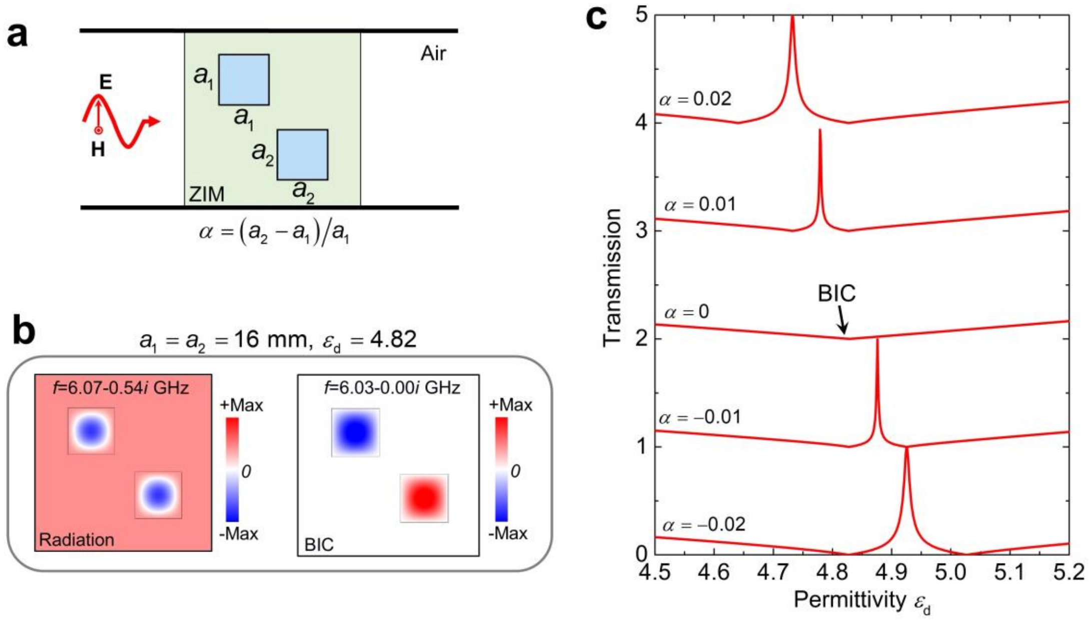

Supplementary Figure 4. Demonstration of BICs in the ZIM with two objects of square shapes. a A two-dimensional (2D) waveguide structure with two dielectric squares embedded in the ZIM. The lengths of two squares are a_1 and a_2, respectively. The asymmetry parameter is defined as $\alpha = (a_2 - a_1)/a_1$. **b** Eigenmode analysis with $a_1 = a_2 = 16$ mm and $\varepsilon_d = 4.82$. A symmetry mode and an antisymmetric mode were found around the frequency of 6.05 GHz. **c** Transmission vs ε_d for different α. For $\alpha \to 0$, the transmission peak becomes sharper. In the case of $\alpha = 0$, the EIT window is disappeared from the transmission spectrum. This is typical signature of a BIC mode manifested by a resonance with zero linewidth. In numerical calculations, $a_1 = 16$ mm and the working frequency is 6.05 GHz.

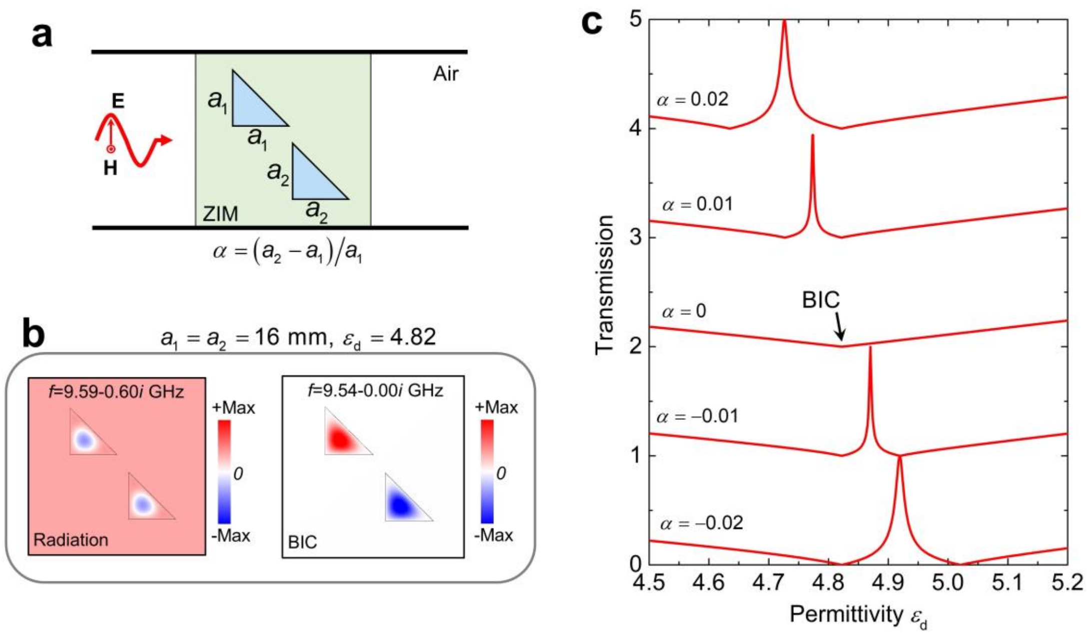

Supplementary Figure 5. Demonstration of BICs in the ZIM with two objects of triangular shapes.

a A two-dimensional (2D) waveguide structure with two triangular dielectrics embedded inside the ZIM. The side lengths of two triangles are a_1 and a_2, respectively. The asymmetry parameter is defined as $\alpha = (a_2 - a_1)/a_1$. **b** Eigenmode analysis with $a_1 = a_2 = 16\ \text{mm}$ and $\varepsilon_d = 4.82$. A symmetry mode and an antisymmetric mode were found around the frequency of 9.56 GHz. **c** Transmission *vs* ε_d for different α. In numerical calculations, $a_1 = 16\ \text{mm}$ and the working frequency is 9.56 GHz.

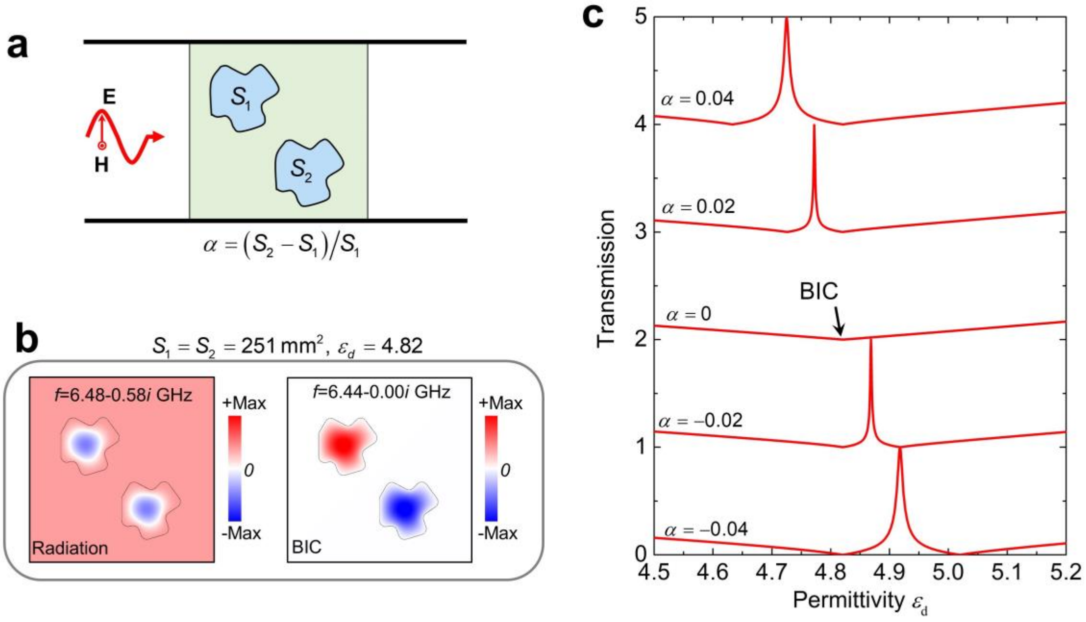

Supplementary Figure 6. Demonstration of BICs in the ZIM with two objects of arbitrary shapes

a Two identical dielectric objects of arbitrary shape embedded inside the ZIM. The areas of dielectric are S_1 and S_2, respectively. The asymmetry parameter is defined as $\alpha = (S_2 - S_1)/S_1$. **b** Eigenmode analysis with $S_1 = S_2 = 251\,\text{mm}^2$ and $\varepsilon_\text{d} = 4.82$. A symmetry mode and an antisymmetric mode were found around the frequency of 6.46 GHz. **c** Transmission vs ε_d for different α. In numerical calculations, $S_1 = 251\,\text{mm}^2$ and the working frequency is 6.46 GHz.

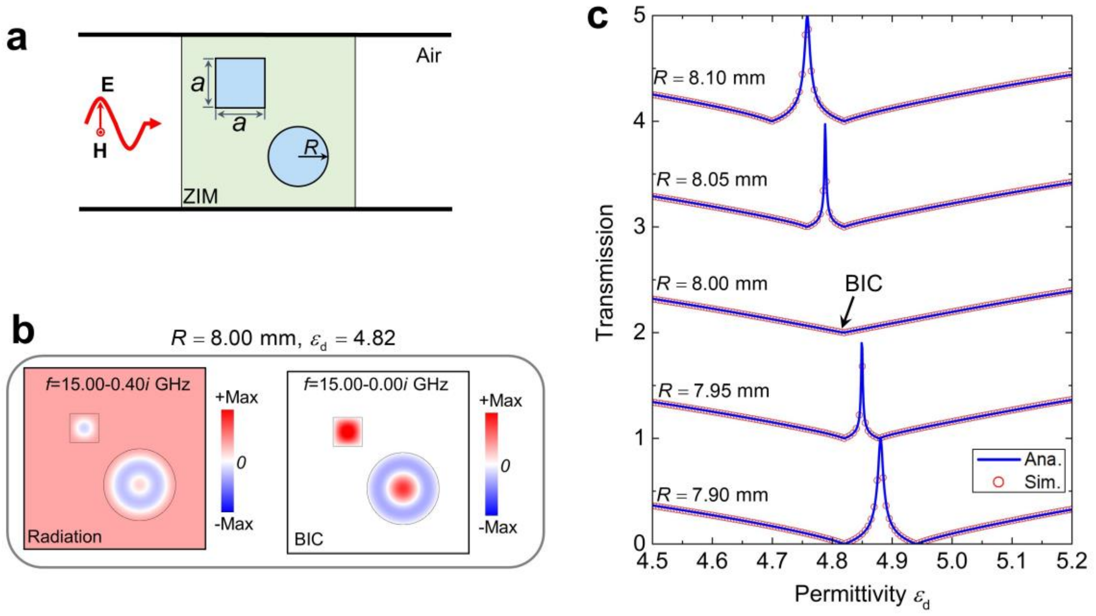

Supplementary Figure 7. Demonstration of BICs in the ZIM with a square-shaped object and a cylindrical object. a A waveguide with a ZIM that contains a square-shaped object and a cylindrical object. The length of the square is *a*=6.437 mm, and the radius of cylinder is denoted as *R*. **b** Eigenmode analysis with *a*=6.437 mm, *R*=8.00 mm, and $\varepsilon_d = 4.82$. There is no symmetry between the two objects but we still find a symmetry mode and an antisymmetric mode (BIC) at the 15 GHz. **c** Transmission vs ε_d for different R. The blue lines are analytical results, and the red circles show the results based on numerical simulations. In simulations, the working frequency is fixed at 15 GHz. A zero linewidth of transmission is found at *R*=8.00 mm and $\varepsilon_d = 4.82$, which indicates a BIC mode here.

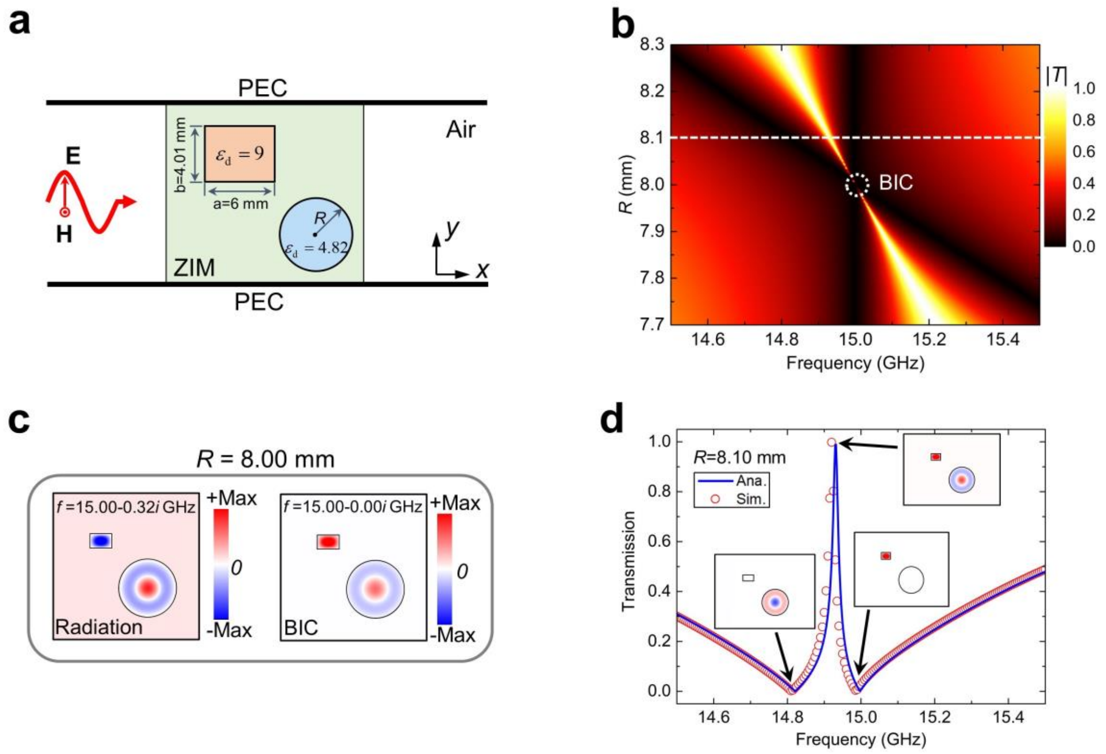

Supplementary Figure 8. Geometric-symmetry-free and materials-independent BIC in frequency domain. **a** A rectangular object with permittivity $\varepsilon_{\mathrm{d}} = 9$ and a cylindrical one with permittivity $\varepsilon_{\mathrm{d}} = 4.82$ embedded inside non-dispersive ZIM. The length and width of the rectangular object are *a*=6 mm and *b*=4.01 mm, respectively. The radius of the cylindrical object is *R*. **b** Transmission as a function of frequency and the radius of cylinder. An ideal BIC occurs at *f* =15 GHz and *R* = 8 mm (the white dash circle). **c** Eigenmode analysis for a rectangular object and a cylindrical object embedded in ZIM environment. The field patterns are the distributions of out-of-plane magnetic field. The radius of cylinder is *R* = 8 mm. **d** Transmission spectrum for a quasi-BIC with *R*=8.10 mm. Insets show the magnetic field distributions inside the ZIM region at the first transmission dip, the transmission peak, and the second dip. The solid line shows analytical results, and the circles are for numerical simulations. Analytical results agree well with simulated results each other.

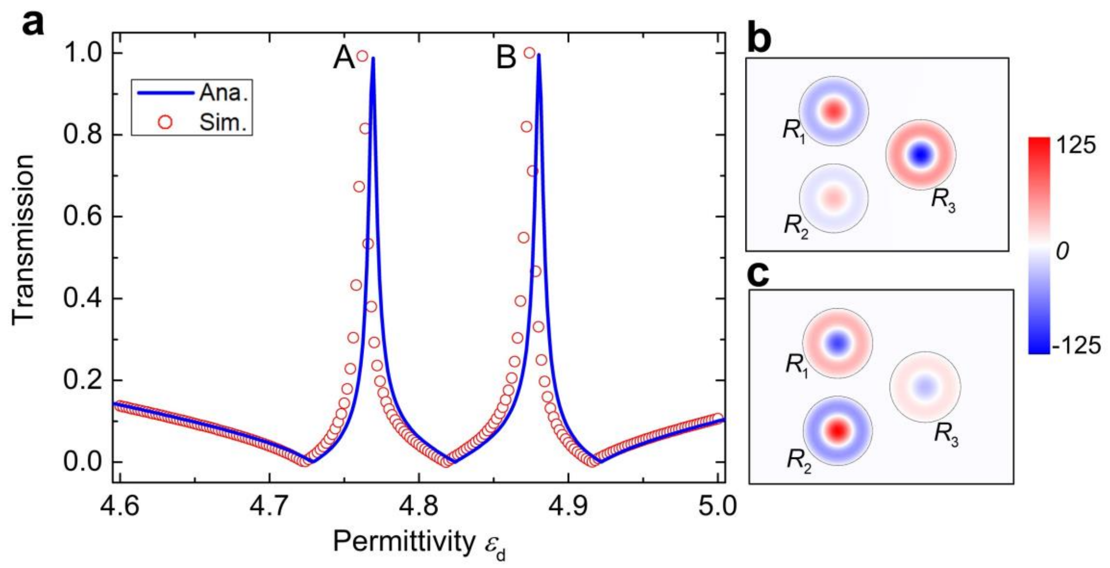

Supplementary Figure 9. Demonstration of BICs in the ZIM with *N*=3 objects of cylindrical shapes. **a** Transmission vs ε_d for the case of *N*=3. The blue solid curve and red circles represent analytical and simulated results, respectively. In calculations, the working frequency is 15 GHz, $R_1 = 8\ \text{mm}$, $R_2 = 7.92\ \text{mm}$ and $R_3 = 8.08\ \text{mm}$. Here $\alpha_2 = (R_2 - R_1)/R_1 = -0.01$ and $\alpha_3 = (R_3 - R_1)/R_1 = 0.01$. **b** and **c** are out-of-plane magnetic field distributions at transmission peak A and peak B, respectively.

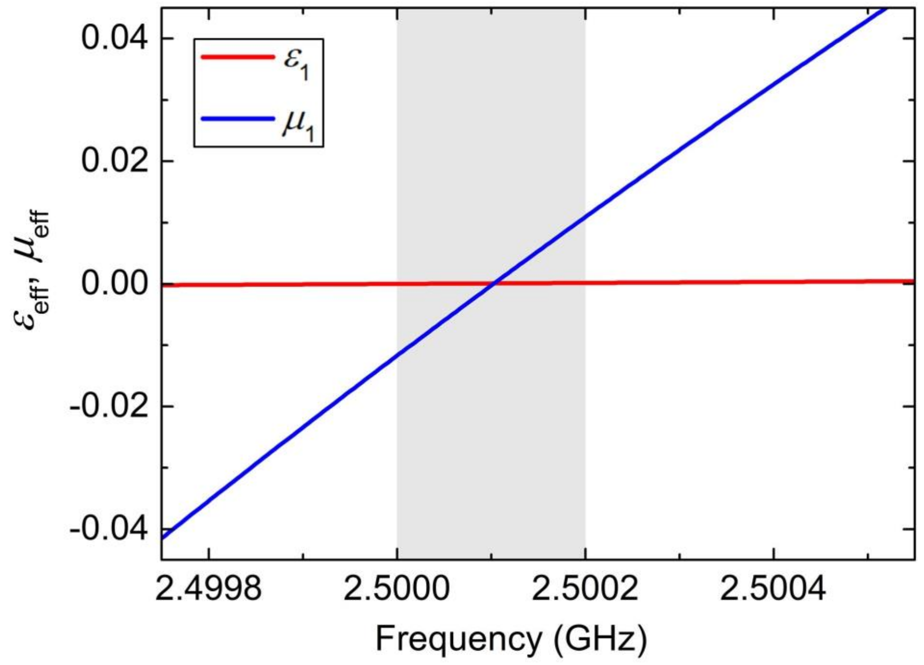

Supplementary Figure 10. Analytical prediction of the dispersion of effective permittivity and permeability of waveguide junction. The red curve and blue curve represent ε_{eff} and μ_{eff}. Although ε_{eff} changes slowly with frequency, μ_{eff} changes quickly with frequency, leading to an extremely narrow ZIM window. Considering the problem of accuracy in simulation, we set $R_0 = 14.0848$ mm in theoretical calculations.

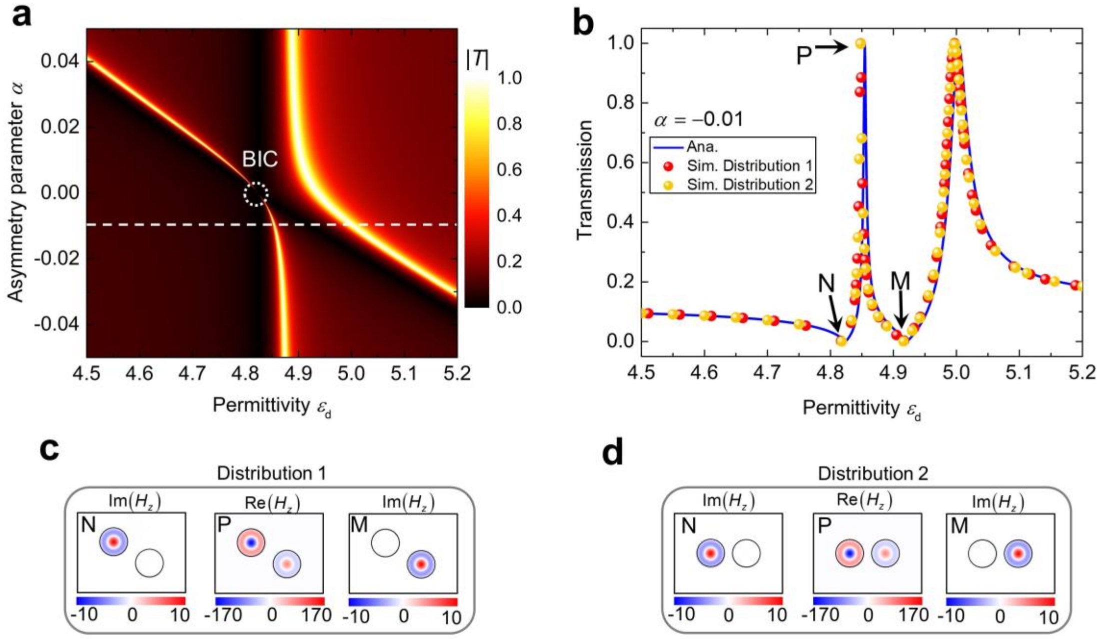

Supplementary Figure 11. BIC illustrations in ENZ-based host. a Analytical 2D map of transmission as a function of ε_d and asymmetry parameter α. The relevant parameters are as follows: R_1=8 mm, w = 44 mm, l = 60 mm and the working frequency is 15 GHz. **b** Transmission spectrum for $\alpha = -0.01$. The blue solid curve represents the analytical results. The red balls and yellow balls represent the simulated results for the two different distributions. Simulated magnetic field distribution patterns of distribution 1 and distribution 2 are shown in **c** and **d**, respectively. N, M, and P corresponding to two transmission valleys and the peak, respectively. In simulations, the incident magnetic field is 1 A m^{-1} and ENZ is set as $\varepsilon_1 = 10^{-4}$ and $\mu_1 = 1$.

Supplementary References

1. Wu, Y. & Li, J. Total reflection and cloaking by zero index metamaterials loaded with rectangular dielectric defects. *Appl. Phys. Lett.* **102,** 183105 (2013).

2. Giovampaola, C. D. & Engheta, N. Plasmonics without negative dielectrics. *Phys. Rev. B* **93,** 195152 (2016).

3. Liberal, I., Mahmoud, A. M., Li, Y., Edwards, B. & Engheta, N. Photonic doping of epsilon-near-zero media. *Science* **355,** 1058-1062 (2017).

4. Xu, Y. & Chen, H. Total reflection and transmission by epsilon-near-zero metamaterials with defects. *Appl. Phys. Lett.* **98,** 113501 (2011).
